# Social vulnerability of the people exposed to wildfires in U.S. West Coast states

**DOI:** 10.1126/sciadv.adh4615

**Published:** 2023-09-20

**Authors:** Arash Modaresi Rad, John T. Abatzoglou, Erica Fleishman, Miranda H. Mockrin, Volker C. Radeloff, Yavar Pourmohamad, Megan Cattau, J. Michael Johnson, Philip Higuera, Nicholas J. Nauslar, Mojtaba Sadegh

**Affiliations:** ^1^ Department of Civil Engineering, Boise State University, Boise, ID, USA.; ^2^ Management of Complex Systems Department, University of California, Merced, CA, USA.; ^3^ College of Earth, Ocean, and Atmospheric Sciences, Oregon State University, Corvallis, OR, USA.; ^4^ Northern Research Station, U.S.D.A. Forest Service, Baltimore, MD, USA.; ^5^ SILVIS Lab, Department of Forest Ecology and Management, University of Wisconsin-Madison, Madison, WI, USA.; ^6^ Human-Environment Systems, Boise State University, Boise, ID, USA.; ^7^ Lynker Technologies LLC, Fort Collins, CO, USA.; ^8^ Department of Ecosystem and Conservation Sciences, University of Montana, Missoula, MT, USA.; ^9^ Bureau of Land Management, Boise, ID, USA.; ^10^ United Nations University Institute for Water, Environment and Health, Hamilton, ON, Canada.

## Abstract

Understanding of the vulnerability of populations exposed to wildfires is limited. We used an index from the U.S. Centers for Disease Control and Prevention to assess the social vulnerability of populations exposed to wildfire from 2000–2021 in California, Oregon, and Washington, which accounted for 90% of exposures in the western United States. The number of people exposed to fire from 2000–2010 to 2011–2021 increased substantially, with the largest increase, nearly 250%, for people with high social vulnerability. In Oregon and Washington, a higher percentage of exposed people were highly vulnerable (>40%) than in California (~8%). Increased social vulnerability of populations in burned areas was the primary contributor to increased exposure of the highly vulnerable in California, whereas encroachment of wildfires on vulnerable populations was the primary contributor in Oregon and Washington. Our results emphasize the importance of integrating the vulnerability of at-risk populations in wildfire mitigation and adaptation plans.

## Teaser

Number of socially vulnerable people exposed to wildfires surged in recent decade with different regional patterns and drivers.

## INTRODUCTION

The frequency of climate-related extreme events has increased substantially in the past several decades (*1*), with unequal burdens among human communities (*2*). These unequal impacts stem from a range of social, economic, and institutional factors, which affect populations’ exposure to—and ability to cope with—disasters (*2*–*4*). The traditional hazard paradigm, however, primarily focuses on the biological and physical drivers of extreme events and largely disregards the social characteristics of exposed populations (*5*). This paradigm often leads to top-down, hierarchical solutions for mitigating the adverse effects of hazards (*6*). By contrast, the vulnerability paradigm focuses on the social, economic, and demographic factors that turn extreme events into catastrophes (*7*, *8*). The latter paradigm views hazards as natural, but not inherently disasters, and attributes the occurrence of disasters to the mismatch among the biological and physical environment, built environment, and social systems (*6*, *9*).

Wildfire—hereafter fire—is a climate-related extreme that, in recent decades, has increasingly affected many regions worldwide, including the western United States (*10*–*12*). Over the past two decades, the number of people in the western United States that lived within fire-affected areas increased by 185% (*13*), and wildfire-caused home and structure losses increased by 246% (*12*). However, the landscape of social vulnerability—the degree of social, economic, and demographic susceptibility to harm from a hazard (*14*, *15*)—of the population exposed to fire is not well known (*16*–*21*). Information about social vulnerability is key to mitigate losses and ensure equitable and effective fire recovery and anticipatory planning (*8*, *18*, *22*). The baseline of social vulnerability is generally lower in the wildland-urban interface, despite a higher fire risk (*23*, *24*); however, this does not apply to certain populations, for example, those living on Native American reservations (*24*). Nevertheless, highly vulnerable populations often are disproportionately exposed to fire, for example, in California (*25*, *26*), Spain (*27*), Portugal (*28*), and Australia (*29*).

Differential access to social, political, and economic resources affects the ability of individuals and communities to mitigate and adapt to fire (*18*, *24*, *30*). This includes resources to reduce the likelihood of home loss (e.g., by reducing flammable materials around structures and home hardening), ability to respond during a fire (e.g., by evacuating elderly and disabled people and by providing effective, accessible emergency messages), and ability to recover after a fire (e.g., insurance coverage and resources to rebuild a home). Furthermore, the traditional paradigm of cost-benefit analysis in fire danger mitigation programs preferentially allocates resources to those in higher socioeconomic strata (e.g., those with higher property values), who generally have lower social vulnerability (*31*). In contrast, those with higher social vulnerability generally experience disproportionate impacts from disasters and may never recover (*32*). This warrants a comprehensive, large-scale study of the social vulnerability of populations exposed to fire (*4*, *33*).

Here, we examined the social vulnerability of people exposed to fires—i.e., lived within areas recently burned—from 2000–2021 in California, Oregon, and Washington through the lens of social vulnerability. These three states accounted for 90% of population exposures to fires in the western United States in the past two decades (*13*). We define vulnerability as a social condition that exists before fire occurrence (*34*) and is driven by historical social, economic, political, and institutional factors (*6*). Here, vulnerability is not dependent on the biological and physical drivers of fire (*24*). We asked four questions.

1) Were highly vulnerable people disproportionately exposed to fire? Previous studies indicated disproportionate exposure of the most vulnerable populations (*26*, *28*, *29*). However, the variability of this unbalanced exposure across large spatial extents remains largely uncharacterized.
2) How did the vulnerability of exposed populations change over the past two decades? Previous studies generally viewed fire exposure as static (*23*, *27*) and did not explore changes in the vulnerability of exposed populations over time.
3) Did trends in population characteristics before fire alter the vulnerability of people exposed to fire? The drivers of changing fire impacts on socially vulnerable populations are not known.
4) Is the social vulnerability of the fire-exposed and unexposed populations in each state equal?

To answer these questions, we integrated annual fire perimeters from the U.S. National Interagency Fire Center (NIFC) from 2000–2021 (*35*), gridded annual population data (92-m resolution) from WorldPop (*36*), and the social vulnerability index (SVI) (*32*, *34*) at the census tract level from the U.S. Centers for Disease Control and Prevention (CDC). We defined fire exposure as a residence within a fire perimeter, and we classified social vulnerability on the basis of the SVI before the fire. Unless otherwise noted, we use the term vulnerability to indicate social vulnerability. SVI has multiple measures in a nested hierarchy, of which we used the overall SVI, its four dimensions or themes (socioeconomic status, household composition and disability, minority status and language, and housing type and transportation), and a selected set of subdimensions that we believed to be most relevant to populations’ capacity to cope with a fire (see fig. S1). We classified SVI as low (≤0.25), medium-low (0.25 < SVI ≤ 0.5), medium-high (0.5 < SVI ≤ 0.75), or high (>0.75).

## RESULTS

### Co-occurrence of social vulnerability and fire exposure

**Fig. 1.**
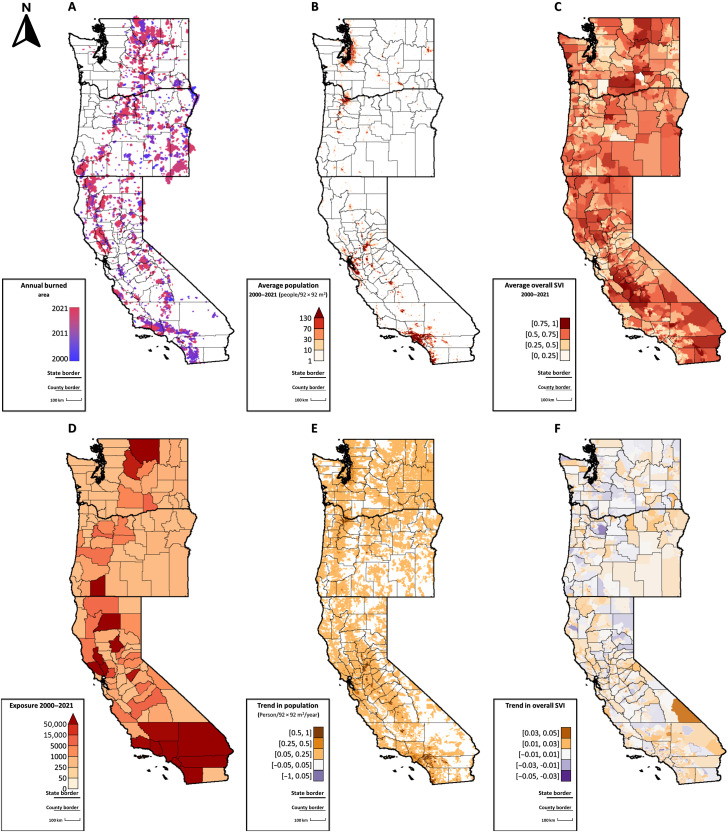
Co-occurrence of fires, human population, and social vulnerability. (**A**) Fire perimeters by year. (**B**) Average population in each 92-m grid cell from 2000–2021. (**C**) Average value of the overall SVI in each grid cell. (**D**) Total number of people exposed to fire from 2000–2021 in each county. (**E**) Population trends in each grid cell (people per year). (**F**) Trends in overall social vulnerability in each grid cell (SVI per year).

Nearly half a million (494,554) people were directly exposed to fire in the West Coast states from 2000–2021 (data S1 to S11). The overwhelming majority of these people (91.5%) were in California, with less than 5% of the exposed population in either Oregon or Washington (Fig. 1D). For reference, California accounted for 77.9% of the three states’ total population and 52.6% of the burned area from 2000–2021, whereas Oregon and Washington accounted for 8.0 and 14.1% of the total population and 29.8 and 17.8% of the burned area, respectively (data S1 to S11). Exposure per unit area burned also was greater in California (5.95 people/km^2^) than in Oregon (0.51 people/km^2^) or Washington (0.78 people/km^2^). Most exposures occurred in northern and coastal southern California, west-central Oregon, and central Washington (Fig. 1D).

**Fig. 2.**
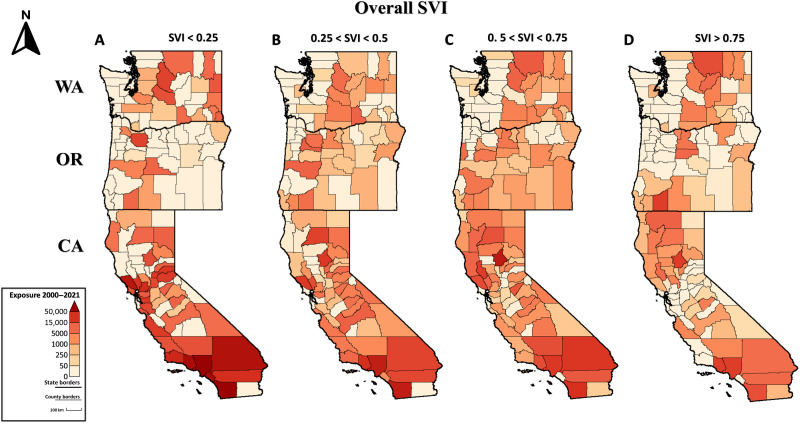
County-level cumulative fire-exposed population from 2000–2021. (**A** to **D**) Number of exposures in four social vulnerability classes. See fig. S2 for exposures relative to social vulnerability dimensions.

In all three states, roughly half of the exposed people had medium-low or medium-high social vulnerability (Fig. 2). However, the percentage of exposed people with high vulnerability diverged widely among the states. Highly vulnerable people accounted for 8.1% of exposures yet 35.1% of California’s general population, compared with 45.4% of exposures (23.5% of the population) and 43.8% of exposures (17.7% of the population) in Oregon and Washington, respectively (data S1 to S11).

Fire exposures across the social vulnerability spectrum followed a distinct spatial pattern within states (Fig. 2). The majority of people exposed in central California had medium vulnerability, whereas the majority of people exposed in northern California had medium-high or high vulnerability. There also were clear coast-to-inland and north-to-south vulnerability gradients in California: Exposed people in the central to south coast had low vulnerability, whereas those in the interior northern part of the state had higher vulnerability (Fig. 2). Fire incidence, and therefore fire exposure, was generally low in wet forests of coastal Oregon and Washington (Figs. 1A and 2). Across Oregon, most exposed people had medium vulnerability, whereas fires in a handful of counties in southern and north-central Oregon disproportionately exposed highly vulnerable people. In Washington, most of the exposures of people with medium-high and high vulnerability occurred in the center of the state (Fig. 2, C and D). Refer to figs. S2 and S3, section S1, and tables S1 to S3 for additional information about exposures relative to vulnerability dimensions.

### Inequality of fire exposure

**Fig. 3.**
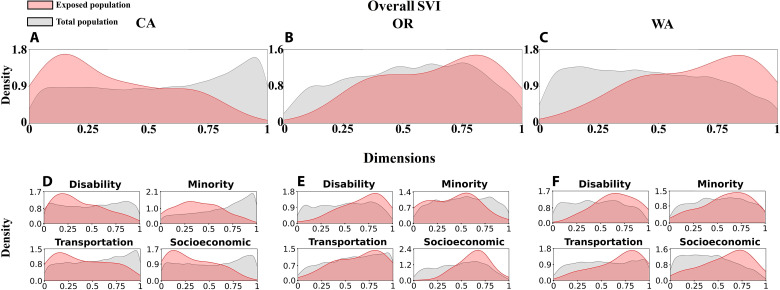
Inequality of fire exposure. (**A** to **C**) Distribution of overall social vulnerability (0, low; 1, high) among the fire-exposed population (pink) and the state population (gray) in California (left column), Oregon (central column), and Washington (right column). (**D** to **F**) Distributions of dimensions of social vulnerability in each state. Socioeconomic, socioeconomic status; disability, household composition and disability; minority, minority status and language; transportation, housing type and transportation (also see fig. S1).

A few key social vulnerability characteristics of the people exposed to fires were common among the West Coast states (Fig. 3). For example, exposed people tended to be associated with higher vulnerability in terms of residing in mobile homes and being 65 years old or older (fig. S4). Important differences were also evident. Overall SVI of the people exposed to fires was lower in California (Fig. 3A) than in Oregon and Washington (Fig. 3, B and C).

Vulnerability distributions of fire-exposed people differed considerably from those of the total population in all three states (Fig. 3, A to C, and tables S4 to S6). The overall SVI distribution of the exposed population was skewed opposite that of the state population in California (lower overall vulnerability of the exposed population; Fig. 3A) and Washington (higher overall vulnerability of the exposed population; Fig. 3C), whereas the difference between the SVI distributions of the exposed and state population was markedly smaller in Oregon (Fig. 3B). The distributions of the different dimensions of vulnerability of the exposed and total population were generally similar to those of the overall SVI in California and Washington (Fig. 3, D and F), but not in Oregon (Fig. 3E). In Oregon, the minority status and language dimension skewed toward lower vulnerability for the exposed than the background-state population, whereas the socioeconomic status, housing type and transportation, and household composition and disability dimensions indicated higher vulnerability for the exposed populations. In California, the distributions of exposed populations living in poverty and with a disability were skewed lower than those of the state population, whereas the skews were reversed in Oregon and Washington (fig. S4). In California and Oregon, the proportion of the population exposed to fire who spoke English less than well generally was lower than that of the state population, whereas a disproportionately high proportion of those who spoke English less than well were exposed to fires in Washington (fig. S4).

### Increasing burned area and population exposure to fires

Total fire area, including unburned and reburned patches within fire perimeters, increased by 116% in the West Coast states from 2000–2010 to 2011–2021, with an increase of 99% in California, 111% in Oregon, and 197% in Washington (Table 1). Summed over the 22 years, these burned areas represent 18.8, 17.3, and 14.7% of the land surface area of California, Oregon, and Washington, respectively. The number of people exposed to fire increased by 27% across the three states in the past two decades, including relative increases of 13% in California, 925% in Oregon, and 366% in Washington (Table 1 and data S1 to S11).

**Table 1.** Decadal fire area, population exposed to fire, and total population in West Coast states.

	Cumulative fire area (km^2^)		Population exposed to fire		Increase in number of state residents from 2000–2010 to 2011–2021	Population in 2020
	2000–2010	2011–2021	2000–2010	2011–2021		
All three states	45,663	98,837	217,959	276,595	9.9%	51,480,760
California	25,431	50,606	212,486	240,133	8.7%	39,538,223
Oregon	13,818	29,196	1,960	20,097	12.2%	4,237,256
Washington	6,414	19,035	3,513	16,365	15.1%	7,705,281

### Increasing exposure of socially vulnerable populations

**Fig. 4.**
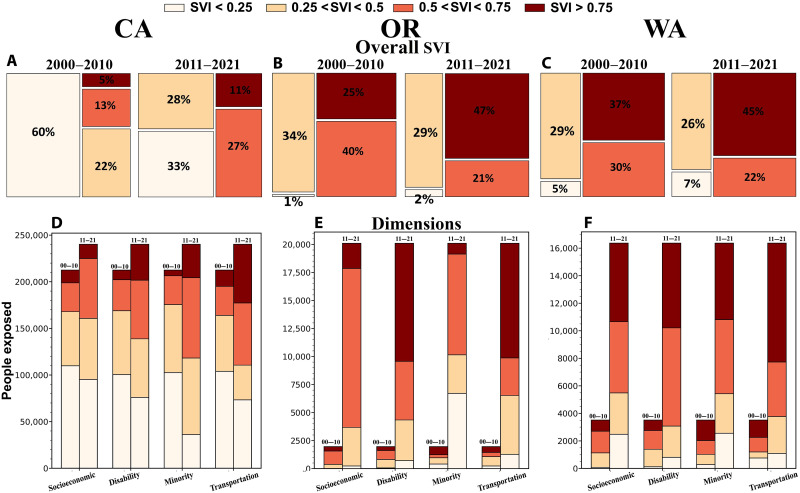
Increasing exposure of socially vulnerable populations to fires. (**A** to **C**) Percentage of exposures to fire by social vulnerability classes from 2000–2010 (left) and 2011–2021 (right) in California (left column), Oregon (middle column), and Washington (right column). (**D** to **F**) Decadal number of exposed people in four social vulnerability classes and each social vulnerability dimension (see fig. S1 for details) by state. Note that *y* axes’ scales differ among (D) to (F).

The number of highly vulnerable people exposed to fire in the West Coast states increased by 249% from the former to latter decade (12,331 people from 2000–2010 and 43,037 people from 2011–2021) (data S1 to S11). The change in the number of highly vulnerable people exposed was greatest in California (10,552 and 26,099 people in the former and latter decades), followed by Oregon (487 and 9526) and Washington (1292 and 7412) (see section S2 for more details). The percentage of the exposed population that was highly vulnerable, however, was much greater in Oregon and Washington than in California (Fig. 4, A to C, and data S1 to S11).

The co-occurrence of fire (Fig. 1A), populated regions (Fig. 1E), and social vulnerability patterns (Fig. 1C) partially explains the trends in vulnerability of exposed people (Fig. 4), but changes in SVI (Fig. 1F) also contributed to increases in exposure of the most vulnerable populations. Social vulnerability decreased across most of the West Coast (Fig. 1F), as it did nationally, but increased in some regions where fires occurred (Fig. 1F), including northwestern and inland southern California, southwestern Oregon, and central Washington.

### Dimensions of vulnerability

The number of people with high vulnerability in all SVI dimensions who were exposed to fire increased in all three states from 2000–2021 (Fig. 4, D and E). Substantial increases (273 to 2944%) in exposure of populations with high vulnerability with respect to household composition and disability and housing type and transportation (Fig. 4, D to F) were consistent among states. However, increases in the exposure of people with high vulnerability in terms of minority status and language were more pronounced in California and Washington than in Oregon (Fig. 4, D to F), and relative increases in exposure of people with highly vulnerable socioeconomic status were greater in Oregon and Washington than in California (Fig. 4, D to F).

### Subdimensions of vulnerability

The number of people exposed to fire who were highly vulnerable in terms of various SVI subdimensions also increased over the past two decades in all three states (fig. S5). The number of people with high vulnerability in the subdimensions of aged 65 years or older and civilian with a disability who was exposed grew considerably in all states (316 and 588% in California, 2469 and 6080% in Oregon, and 1512 and 1187% in Washington, respectively). However, changes in the relative exposure of people with high vulnerability in other SVI subdimensions varied among states. In California, for example, the increase in the exposure of people that spoke English less than well was much greater than in Oregon or Washington. In the latter two states, relative increases in exposure of people with high vulnerability in the below poverty and group quarters subdimensions were substantially greater than those in California (fig. S5).

### Contribution of increasing social vulnerability to increased fire exposure of vulnerable people

To decompose the effect of changes in social vulnerability on the observed increase in the number of exposed people with high vulnerability, we conducted a counterfactual analysis in which we analyzed actual fire perimeters and human population patterns from 2011–2021 but kept the SVI constant at the year 2000 value. By doing so, we evaluated what the social vulnerability of the exposed populations from 2011–2021 would have been if their SVI had not changed since 2000. We selected 2011–2021 for consistency with the analyses described in the previous section.

**Fig. 5.**
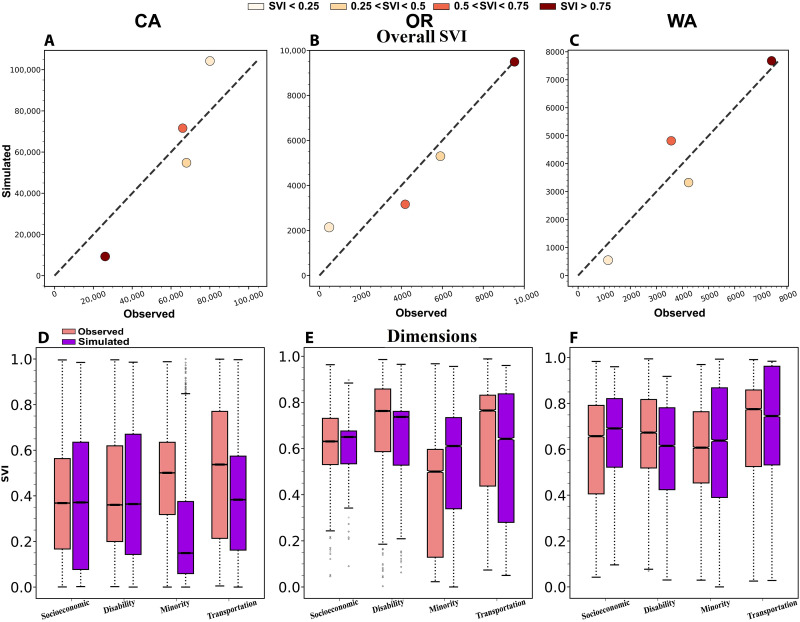
Contribution of social vulnerability trends to increasing vulnerability of exposed people. (**A** to **C**) Number of exposed people from 2011–2021 in each social vulnerability class based on the simulated counterfactual scenario of a static overall SVI after 2000 (*y* axis) and the actual SVI (*x* axis). (**D** to **F**) Social vulnerability (0, low; 1, high) of the exposed population from 2011–2021 in the simulated counterfactual scenario (purple; right) versus observed conditions (pink; left) with respect to each dimension of vulnerability in each state.

Changes in the overall SVI explained much of the increase in the number of highly vulnerable exposed people in California (Fig. 5A), but not in Oregon or Washington (Fig. 5, B and C). In California, SVI changes resulted in an increase in the number of exposed people with high vulnerability (+16,172 people), equivalent to the total increase in the number of highly vulnerable people exposed to fire (Fig. 4A). In contrast, in Oregon and Washington, increases in the number of highly vulnerable exposed people were mainly due to fires increasingly occurring in populated areas with high vulnerability rather than increases in social vulnerability in the fire-exposed regions (Figs. 4, B and C, and 1). The contribution of background SVI dimensions and subdimensions to trends in exposure of highly vulnerable populations was markedly different among states (Fig. 5, D to I). However, background SVI changes in housing type and transportation (dimensions; Fig. 5, D to F) and aged 65 or older (subdimensions; fig. S6) contributed to an increase in the vulnerability of exposed populations in all three states. For more details, see section S3 and tables S7 and S8.

## DISCUSSION

Many national disaster mitigation and prevention programs use cost-benefit analyses to allocate resources, which can skew benefits toward wealthy people (*31*). Disaster-caused monetary losses among affluent people generally exceed those among the less wealthy, but members of the latter group often lose a larger portion of their assets and might never recover from the loss (*34*). Socially vulnerable people may also be more likely to live in hazard-prone areas (*15*). For example, historical socioeconomic and power inequalities have pushed marginalized people to live in areas more prone to floods and in houses that are more likely to incur damage (*31*). Socioeconomic status is the most important driver of vulnerability (*37*, *38*). However, other dimensions of social vulnerability, such as age, minority status, and disability, also affect the ability of subpopulations to cope with disasters (*31*, *39*). Here, we applied a hierarchical model of social vulnerability developed by CDC to assess patterns in the social vulnerability of the populations exposed to fires in California, Oregon, and Washington. Ninety percent of the people exposed to fire in the western United States in the past two decades lived in these three states (*13*). We adopted a conservative definition of fire exposure by analyzing the populations that lived within the boundaries of fires. The number of people indirectly affected by wildfire, such as by wildfire smoke, evacuations, utility disruptions, and road closures, can be several-fold larger.

Our results indicated that the number of highly vulnerable people exposed to fire increased by nearly 250% in the West Coast states from 2000–2010 to 2011–2021. In all three states, the vulnerability of the exposed people increased over the past two decades, a trend that is contrary to national reductions in social vulnerability of the general population (*38*). Our analysis further showed that the increase in social vulnerability, in addition to increased occurrence of fires in regions with high social vulnerability, contributed to the increase in the number of highly vulnerable people exposed to fire.

We also found major differences in the vulnerability of fire-exposed populations among and within the three states. For example, in California, only a small proportion of the exposed people were highly vulnerable—albeit this small proportion translates to a large number—whereas a considerable proportion of the exposed people in Oregon and Washington were highly vulnerable. The vulnerability of people exposed to fires across the urban-rural gradient also differed markedly among states (figs. S7 to S12). For example, a majority of the people with low vulnerability who were exposed in California and Washington were urban dwellers, whereas most of the people with low vulnerability who were exposed in Oregon were rural residents (fig. S7E). In all three states, people with high vulnerability who were exposed to fire lived in both urban and rural areas, but the proportion of exposed, highly vulnerable people who were rural residents was greater in Washington than in California or Oregon (fig. S7H). See sections S4 and S5 and figs. S7 to S12 for more detail.

In all three states, the number of exposed people with high vulnerability in the subdimensions of aged 65 or older and disability increased substantially in the past two decades and constituted a considerable percentage of the total number of people exposed. Seniors may be particularly vulnerable to fire hazards due to diminished sensory abilities, which hamper timely detection of danger, and cognitive impairments that compromise response to fire threats (*40*). For example, 80% of the people killed by the 2018 Camp Fire, the deadliest fire in California’s history, were 65 years of age or older (*41*). Furthermore, the material and informational resources that seniors use and their emergency needs may be different from those of younger generations. As population aging in the United States (*39*) occurs simultaneously with increases in the area burned in a warming climate (*42*), the number of older residents exposed to fires is likely to rise. In addition, people with disabilities are highly vulnerable to fire risks during all phases of fires, from mitigation to evacuation and recovery (*32*, *43*). Thirteen of the 2018 Camp Fire victims, for example, had underlying physical and mental impairments that impeded their ability to evacuate (*41*). Similarly, following evacuations, people with a disability often face major challenges in finding temporary housing that can accommodate their needs (*44*).

The exposure of people with high vulnerability in terms of group quarters (e.g., nursing homes, prisons, and worker dormitories) and limited English-speaking skills increased in all three states and accounted for a notable fraction of all exposures. Evacuation of group quarters requires considerable resources and timely action (*34*). Synchronous severe fire weather and concurrence of major fires in many regions across the western United States (*45*, *46*), however, rapidly exhausts resources for fire suppression and emergency response. These factors, along with the increasing incidence of fires that spread rapidly (e.g., the December 2021 Marshall Fire in Colorado that burned >1000 homes in one day), will necessitate major investments to mitigate fire risks for group quarters and to facilitate systematic evacuations. Furthermore, a majority of fire mitigation and evacuation resources, including warning systems, are in English, which limit their accessibility for non-English speaking residents. Spanish-speaking farmworkers exposed to a fire in eastern Washington in 2014, for example, faced language barriers in receiving evacuation notices from the authorities (*47*).

The social vulnerability of fire-exposed people, compared to state populations as a whole, differed among the three West Coast states. Exposed Californians had lower vulnerability, whereas exposed Washingtonians had higher vulnerability than their state populations. The skew toward higher vulnerability of California’s state population mainly stems from the high vulnerability of people in major metropolitan areas such as Los Angeles and San Francisco, which are not directly exposed to wildfire. Although we did not address indirect effects of fires, vulnerable communities in metropolitan areas often are exposed to unhealthy levels of wildfire smoke (*48*). Air quality in Seattle and San Francisco was the worst among cities worldwide during the peak of fire seasons in recent years (e.g., 2020). Wildfire smoke expands the fire impacts to millions of people who live hundreds to thousands of kilometers away from the burned areas. The range of adverse physical and mental health outcomes of fire is extensive (*49*–*52*) and increases pressure on the health care system (*53*–*55*).

Our findings on the increasing vulnerability of populations exposed to fire have several limitations. First, the gridded population data that we used to estimate exposure to fires may have inaccuracies. However, the population estimates from the U.S. Census and WorldPop were similar. Second, because the finest resolution of the SVI data is the census tract, we had to assign the same SVI value to all residents of a given tract and could not capture within-tract heterogeneity. Furthermore, there are uncertainties in the SVI itself, including the type of variables included and their interdependencies, analysis scale, measurement errors, and data transformation, normalization, weighting, and aggregation; and whether the data represent the entire population (e.g., whether undocumented residents are included and the sample of survey participants in noncensus years) (*14*, *56*). Nevertheless, our study offers important insights about the vulnerability of people exposed to fires.

A majority of fire studies focused on the biological and physical aspects of this hazard and did not address the population characteristics that affect the coping capacity of people exposed to fires (*5*, *13*). Our results indicate strong spatial patterns in highly vulnerable people exposed to fires who may require further assistance (*33*), including both urban and rural residents. Addressing potential social vulnerabilities requires a holistic approach at levels from individual to community to local government. For example, previous literature has shown that affluent urban communities are better positioned to secure federally funded hazard mitigation grants than less-wealthy rural communities (*57*, *58*). Furthermore, broader social and economic forces contribute to rural poverty and isolation, and the likelihood of destructive fires increases with climate change. The vulnerability of rural communities and more generally socially vulnerable populations, therefore, may increase over time by the compounding impacts of fires and deficient adaptation and mitigation investments. This warrants more equitable hazard mitigation efforts.

The U.S. federal government is increasingly including aspects of social vulnerability in administration and design of assistance and hazard reduction programs. For example, the 2021 Infrastructure Investment and Jobs Act created a new Community Defense Wildfire Grants program to assist communities and Tribes in reducing wildfire risk. This program gives preference to low-income or underserved communities and waives financial match requirements for them. However, applications outstripped available funding by a ratio of 3:1 in the first round of applications (*59*). Our findings can contribute to strengthening the efficacy of such programs and informing their expansion at federal, state, and local levels.

The population of the western United States continues to grow and age, and fires in the region are becoming larger and more destructive. These trends will likely increase the number of people exposed to fire, especially those who have high vulnerability in certain dimensions, regardless of overall social vulnerability trends. Furthermore, short-term and long-term displacement due to fires is a source of stress for both the displaced and the recipient populations and can reverberate to the housing market and increase inequities in home ownership and decrease community sustainability (*60*, *61*). Our analysis highlights the need to increase understanding of the social characteristics that affect vulnerability to fire to inform effective mitigation and adaptation strategies (*7*, *8*). Differences in the vulnerability of fire-exposed populations among and within California, Oregon, and Washington suggest a need for community-level fire mitigation, planning, response, and recovery programs (*33*, *62*). Particular attention to residents who are older, living with a disability, living in group quarters, and with limited English-speaking skills may be warranted, and cultural differences need to be addressed for effective policy development and response (*18*, *24*). Our results suggest a need to diversify modes and languages of communication to serve the most vulnerable communities. This emphasis contrasts with the traditional hazard paradigm, which prescribes top-down, engineered solutions that focus mainly on the biological and physical drivers of hazards rather than the social dimensions of vulnerability (*6*).

## MATERIALS AND METHODS

We used fire data from the NIFC, which provides information about individual fires, including perimeters and date, from 2000 to present (*35*). These data are different from the fire perimeter data of the Monitoring Trends in Burn Severity program (*63*): NIFC includes perimeters of all reported fires, whereas Monitoring Trends in Burn Severity program only includes large fires (>400 ha in the western United States). We used annual gridded population data from WorldPop, which uses a machine learning model with a variety of auxiliary variables to disaggregate total reported population at the administrative units (e.g., U.S. census blocks) to grids with 92-m resolution (*36*). Variables that inform population distribution in WorldPop include roads, land cover, built structures, cities or urban areas, night-time lights, infrastructure, environmental data, protected areas, and water bodies (*64*). WorldPop offers annual, global, gridded population density data from 2000 to present. Furthermore, we used the census tract–level SVI from the CDC (*65*). The SVI ranks each tract relative to the national proportion of tracts with equal or lower social vulnerability (*65*). From this hierarchical model of social vulnerability, we used the overall SVI, its four dimensions or themes—socioeconomic status, household composition and disability, minority status and language, and housing type and transportation—and its 15 subdimensions of vulnerability as identified in the 2018 CDC SVI documentation (*65*). SVI data are available for 2000, 2010, 2014, 2016, 2018, and 2020. For each year without a reported SVI, we used the most recent available data. We used SVI terminology as reflected in the 2018 documentation (*65*), which we deemed the most representative of all years. We analyzed data from the years 2000–2021 because they were common among our various data sources.

We conducted all analyses at the census tract level and assigned the same value of the overall SVI and the dimensions and subdimensions of SVI to all people within the tract. We overlaid fire perimeters and the population layer on the census tract perimeters for each year and estimated the total number of people in each tract who lived within the fire perimeters and their SVI. We repeated this analysis for all people in each census tract regardless of exposure. Although the latter information is available from the census, we used WorldPop-derived population in all reported analyses for consistency. WorldPop and census tract populations closely matched. We then used exposure, population size, and associated SVI to estimate distributions of SVI for exposed and unexposed populations. To plot distributions in Fig. 3, we used Gaussian kernel smoothing. For average and trend analyses at the grid cell level (92-m resolution) from 2000–2021, we adopted a mesh grid similar to that of WorldPop, assigned SVI in each census tract in each year to all grid cells encapsulated in the census tract perimeters, and subsequently calculated all statistics. When reporting county and state exposure and general population statistics, we aggregated census tracts encapsulated in each administrative unit. We summarized the SVI data in four classes (quartile level) and estimated exposure and general population statistics for each social vulnerability class.

We used Mood’s median test (*66*) to assess whether the median of the two distributions was significantly different (e.g., SVI distributions of exposed people in the former and latter decades in each state and SVI distributions for exposed and state populations cumulatively in the past two decades; note the difference between population as it relates to humans in the manuscript and its statistical definition). We used a Bartlett test (*66*) to assess whether the variance of two distributions was significantly different and a Mann-Whitney *U* test (*66*) to assess whether the two distributions were similar. We set the significance level for all tests at the 5% (95% confidence level) and reported *P* values for all tests.

## Supplementary Materials

### This PDF file includes:

Sections S1 to S5 Figs. S1 to S13 Tables S1 to S8 Legends for data S1 to S11
