# Supplementary material for: Social vulnerability of the people exposed to wildfires in U.S. West Coast states
Source: Sci Adv. 2023 Sep 20;9(38):eadh4615. doi: 10.1126/sciadv.adh4615 (PMC10511185; doi:10.1126/sciadv.adh4615)
Supplement: Supplementary file 1 — Sections S1 to S5 Figs. S1 to S13 Tables S1 to S8 Legends for data S1 to S11 [file sciadv.adh4615_sm.pdf]

Science Advances
AAAS

Supplementary Materials for

Social vulnerability of the people exposed to wildfires in U.S. West Coast states

Arash Modaresi Rad *et al.*

Corresponding author: Mojtaba Sadegh, mojtabasadegh@boisestate.edu

Sci. Adv. **9**, eadh4615 (2023)
DOI: 10.1126/sciadv.adh4615

The PDF file includes:

Sections S1 to S5
Figs. S1 to S13
Tables S1 to S8
Legends for data S1 to S11

Other Supplementary Material for this manuscript includes the following:

Data S1 to S11

Section S1. Comparison of exposed people with high vulnerability to county population

California was distinct in terms of the discrepancy between counties in which many highly vulnerable people were exposed to fire and counties in which high-vulnerability exposures accounted for a large percentage of the population (Figs. S3A-C). In highly populated Los Angeles and Ventura counties, a large number of individuals with high vulnerability were exposed (~7,500 people in each county), but these people were a marginal percentage of the county population (<1%; Fig. S3A and Table S1). By contrast, the 65 people with high vulnerability who were exposed to fire in Alpine County accounted for 5.5% of the county population (Fig. S3A and Table S1). Counties with low population generally have lower incomes and less access to resources for hazard prevention and recovery (7, 33). In Oregon and Washington, however, the counties in which many people with high vulnerability were exposed were generally the same as those in which a high percentage of the population was exposed to fire (Figs. S3B-C and Tables S2-S3).

Section S2. Percentage of highly vulnerable population in each state that was exposed to fire

The percentage of state residents who had high vulnerability and were exposed to fire increased markedly in all U.S. West Coast states from 2000-2011 to 2011-2021, especially in Oregon (1,607%), where 0.06% and 0.98% of the highly vulnerable population was exposed in the former and latter decades, respectively. In 2020, a year in which the area burned in Oregon was among the greatest on record (67), 0.63% of the state's highly vulnerable population was exposed to fire (Supplementary Data 1-11). The percentages of the highly vulnerable population in California and Washington who were exposed were 0.09% and 0.12% from 2000-2010, respectively, and 0.18% and 0.58% from 2011-2021 (Supplementary Data 1-11).

Section S3. Contribution of increasing social vulnerability to increased exposure of vulnerable populations: Dimensions of vulnerability

Trends in the SVI dimensions of minority status and language in California, and housing type and transportation in California and Oregon (Figs. 5D-E), contributed the most to increases in the social vulnerability of exposed individuals. By contrast, the social vulnerability of exposed populations with respect to minority status and language in Oregon decreased due to SVI trends (Fig. 5E). Changes between counterfactual scenario and observed patterns in other SVI dimensions were minimal, although most were statistically different because sample sizes were large (Table S7).

Trends in the SVI subdimensions of disability and aged 65 or older across all states (Figs. 5G-I), and group quarters and speaks English less than well in California (Fig.

5G), resulted in increases in the social vulnerability of exposed populations. In Oregon, by contrast, trends in the group quarters and speaks English less than well subdimensions reduced the social vulnerability of exposed populations (Fig. 5H). Changes in all subdimensions in all states were statistically significant due to large sample sizes (Table S8), but shifts in some subdimensions (e.g., mobile home and below poverty) were less notable than others (Fig. 5G-I).

Section S4. Increasing exposure of socially vulnerable populations across the rural/urban gradient: Overall social vulnerability

We used the U.S. Decennial Demographic and Housing characteristics data, which were available for 2000, 2010, and 2020, to calculate the percentage of urban residents in each census tract from 2000-2021. For each year, prior decennial data were used. We adopted an arbitrary threshold of 50% to categorize census tracts as urban (≥50% urban residents) or rural (<50% urban residents). Across the three states, nearly 71% of all people exposed were urban residents (Fig. S7). However, the percentage of exposed people who were urban residents varied greatly among states (California: 74%, Oregon: 38%, Washington 30%).

In all states, most exposed people with low vulnerability (overall SVI) were urban residents (urban proportion close to 1) (Fig. S8A), which is dominated by populations exposed in California (i.e., 91.5% of all fire-exposed people). Rural residents who were exposed had medium and high vulnerability (Fig. S8B-D). Further analysis revealed nuanced differences among states, where exposed populations with low vulnerability were more likely to be rural residents in Oregon (Fig. S8E) than in California and Washington (Fig. S8E). People who were exposed and had high vulnerability tended to be more urban residents in California and Oregon, and more rural residents in Washington (Fig. S8H).

The percentage of fire-exposed people with high vulnerability (overall SVI) increased from 2000-2010 to 2011-2021 across the study region (Fig. S9), with a more pronounced increase (four-fold) among urban populations (Figs. S9C-D). This trend generally was consistent at the level of states (Fig. S10), with the exception of rural exposures in Washington (Fig. S10E-F). However, the percentage of exposed urban and rural residents with high vulnerability was different in California than in Oregon and Washington (Fig. S10). In Oregon, a large percentage of the urban population exposed to fire had high vulnerability, and this percentage increased markedly over the past two decades (Fig. S10I-J), from 66% to 94% (Fig. S10J). In contrast, only 12% of the urban population exposed to fire in California in the recent decade had high social vulnerability (Fig. S10H).

Section S5. Increasing exposure of socially vulnerable populations across the rural to urban gradient: Dimensions of social vulnerability

Exposure of both urban and rural populations with high social vulnerability generally increased from 2000-2010 to 2011-2021 and across the study region (Fig. S11). The exception was urban populations with vulnerable socioeconomic status, which declined by 35% over the past two decades (Fig. S11B). Furthermore, the number of exposed people with high social vulnerability in various dimensions differed between urban and rural populations, especially in the recent decade (Fig. S11). In the recent decade, exposures of rural populations with high vulnerability in the socioeconomic status dimension increased, and exposures of rural populations with high vulnerability in the minority status and language dimension decreased (Fig. S11A). However, exposures of urban populations with high vulnerability in the housing type and transportation dimension increased, and exposures of urban populations with high vulnerability in the socioeconomic status dimension decreased (Fig. S11B). Distributions of urban proportion for the exposed populations in various social vulnerability classes and dimensions (Figs. S12A-D) and subdimensions (Figs. S12E-H) were consistent with these results.

The number of highly vulnerable people, both urban and rural, exposed to fire generally increased over the past two decades across the study region, with several notable differences among states and social vulnerability dimensions (Fig. S13). In California, exposure of urban populations with high vulnerability in the socioeconomic status dimension declined markedly (-57%; Fig. S13D); and exposure of rural populations with high vulnerability in household composition and disability dimension declined (-6%; Fig. S13A). Additionally, in Oregon, exposures of urban populations with high social vulnerability in the minority status and language dimension decreased (-23%; Fig. S13E).

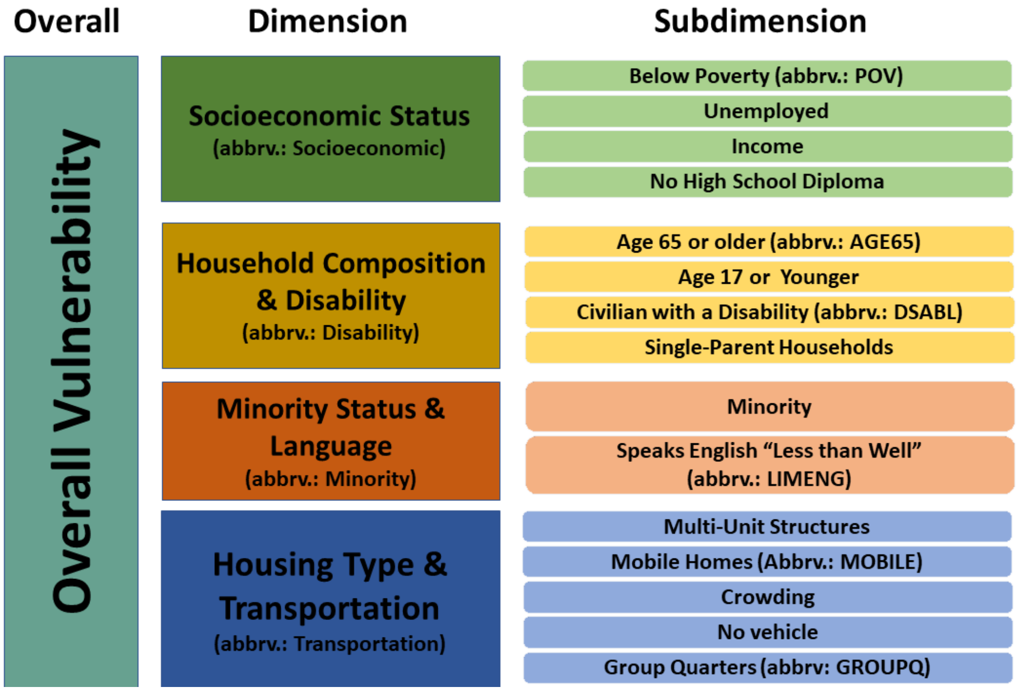

Figure S1. Social vulnerability and its dimensions and subdimensions. We adopted the U.S. Centers for Disease Control and Prevention's hierarchical model of vulnerability as described in its 2018 documentation.

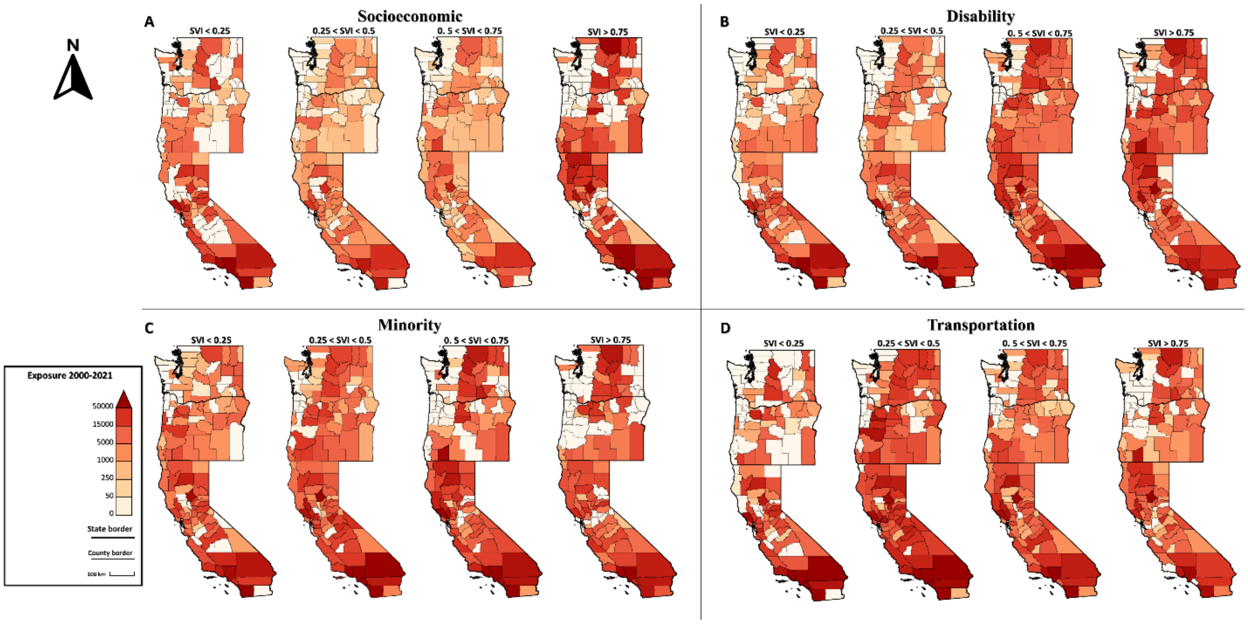

Figure S2. County-level population exposed to fire from 2000-2021 in four social vulnerability classes with respect to dimensions of vulnerability.

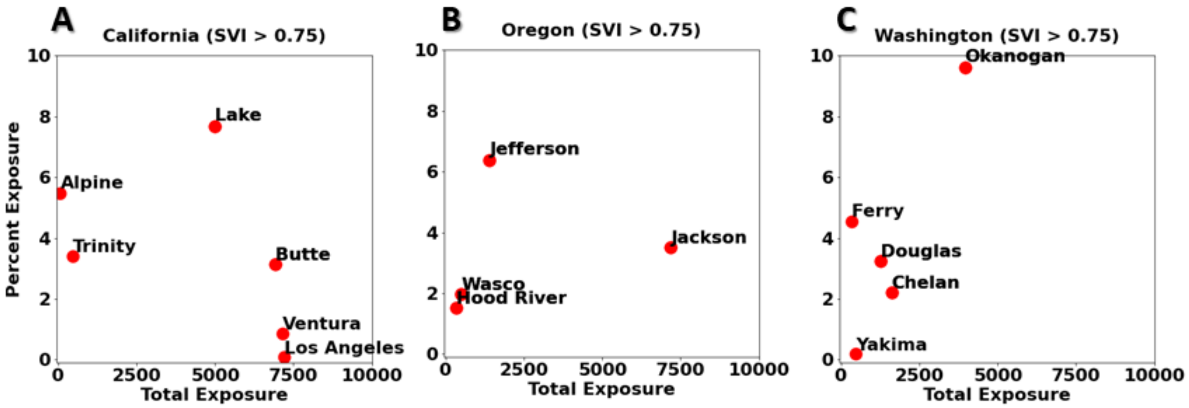

Figure S3. Normalized county-level cumulative fire-exposed population from 2000-2021. **(A-C)** The four counties in each state with the greatest number of exposures of highly vulnerable individuals (x-axis) and the four counties with the greatest percentage of population exposed to fire (y-axis). Overlap of the two ranks can result in display of fewer than 8 counties.

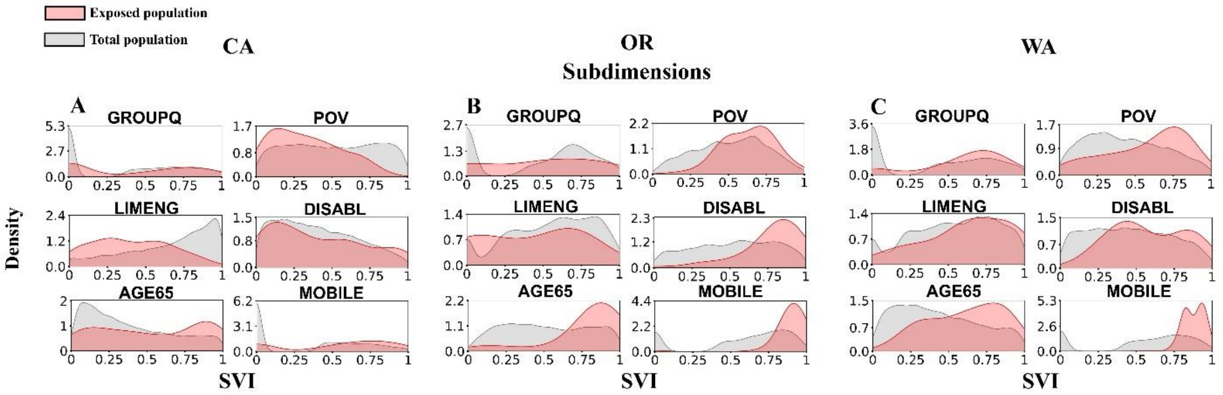

Figure S4. Inequality of fire exposure. (A-C) Distribution of selected subdimensions of social vulnerability (0: low – 1: high) among the fire-exposed population (pink) and the state population (gray) in California (CA; left column), Oregon (OR; central column), and Washington (WA; right column). Abbreviations: GROUPQ: group quarters, POV: below poverty, LIMENG: speaks English less than well, DSABL: civilian with a disability, AGE65: aged 65 or older, MOBILE: mobile homes (also see Fig. S1).

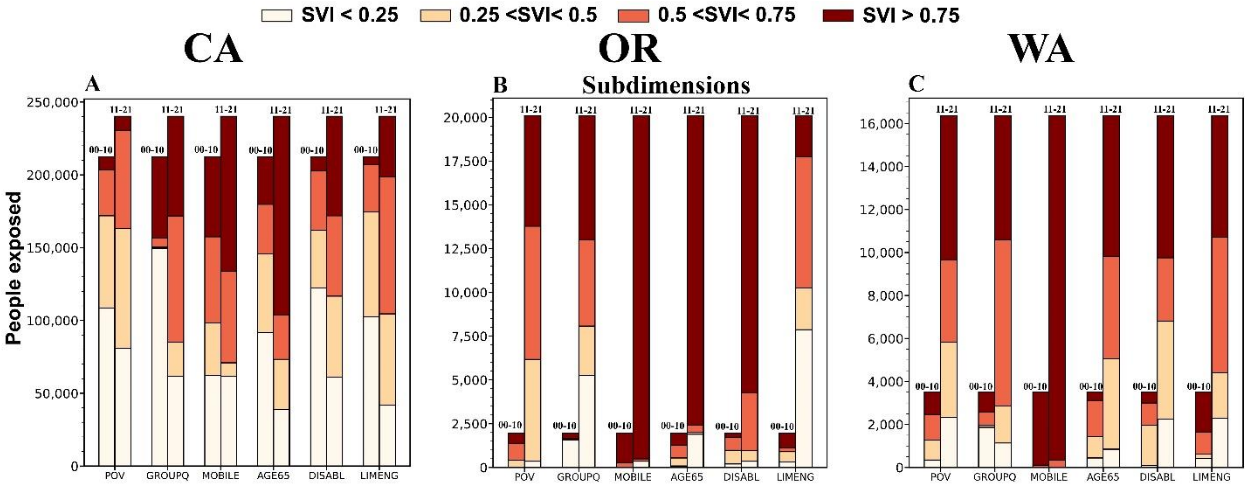

Figure S5. Increasing exposure of socially vulnerable populations to fires. (A-C) Decadal number of exposed people in four social vulnerability classes and six social vulnerability subdimensions by state. Note that y-axes' scales differ among panels.

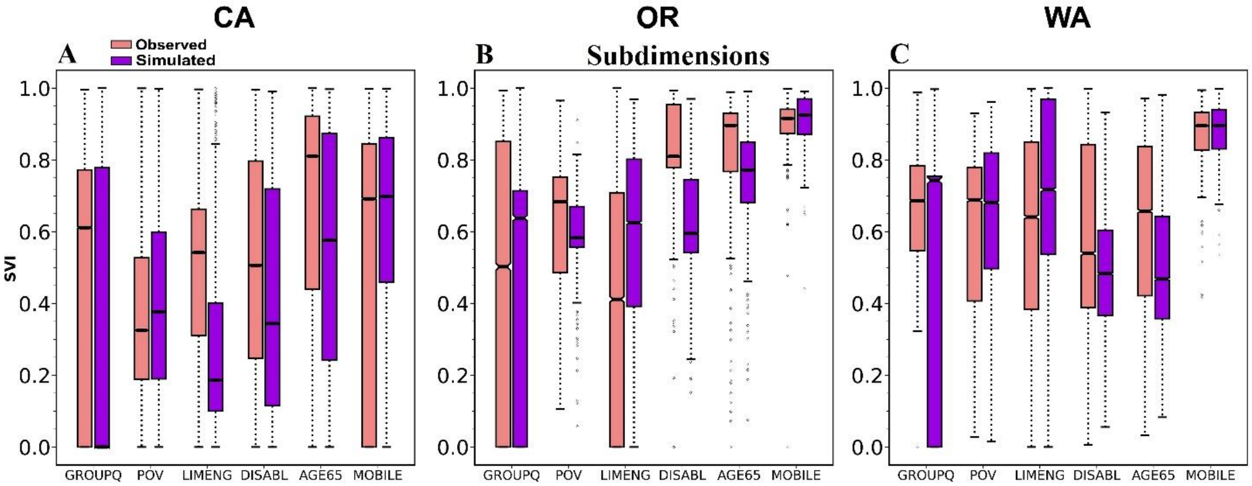

Figure S6. Contribution of social vulnerability trends to increasing vulnerability of exposed people. (A-C) Social vulnerability (0: low – 1: high) of the exposed population from 2011-2021 in the counterfactual scenario (purple; right) versus observed conditions (pink; left) with respect to each subdimension of vulnerability in each state.

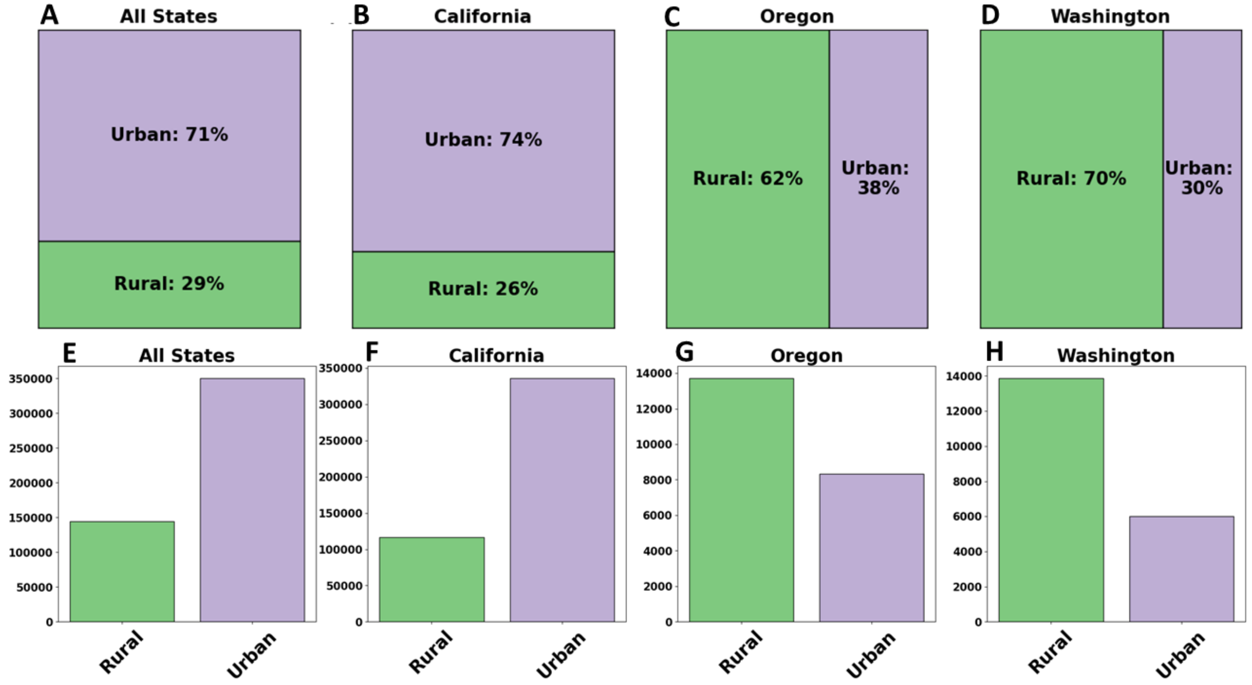

Figure S7. Population exposure to fire in urban and rural areas. (A-D) Percentage of people exposed to fire who were urban and rural residents across the region (A) and in each state (B-D). (E-F) Number of people exposed to fire who were urban and rural residents across the study region (E) and in each state (F-H).

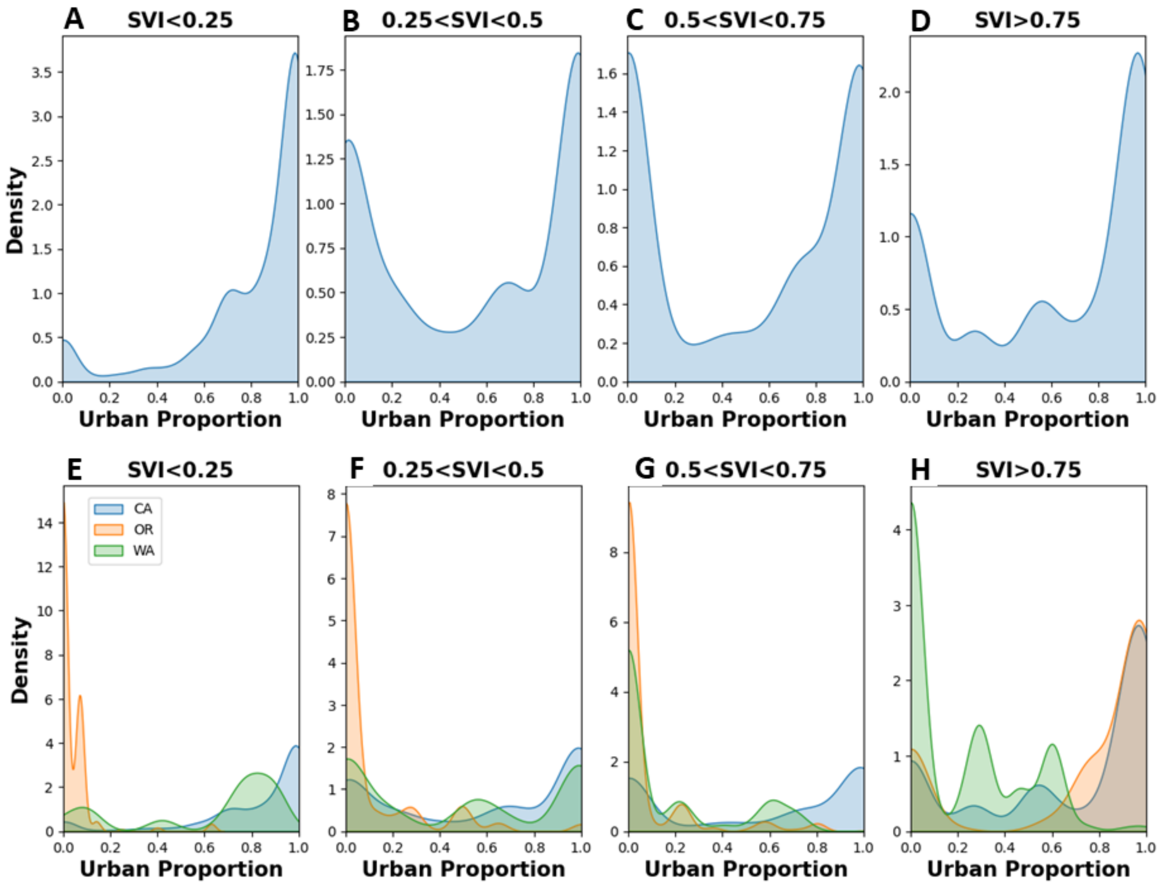

Figure S8. Distribution of urban proportion for exposed population in each social vulnerability class across the study region (top; A-D) and in each state (bottom; E-H). Urban proportion is defined as the proportion of urban residents in each census tract.

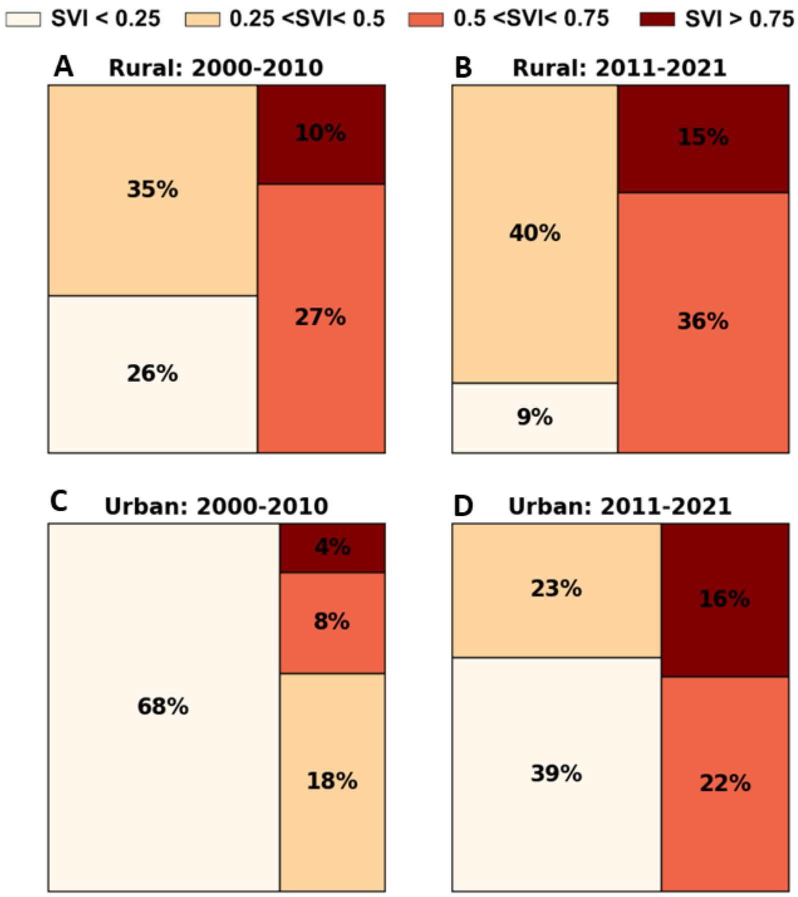

Figure S9. Percentage of exposures to fire by social vulnerability class from 2000-2010 (left; A,C) and 2011-2021 (right, B,D) in rural (top; A,B) and urban (bottom; C,D) areas across the study region.

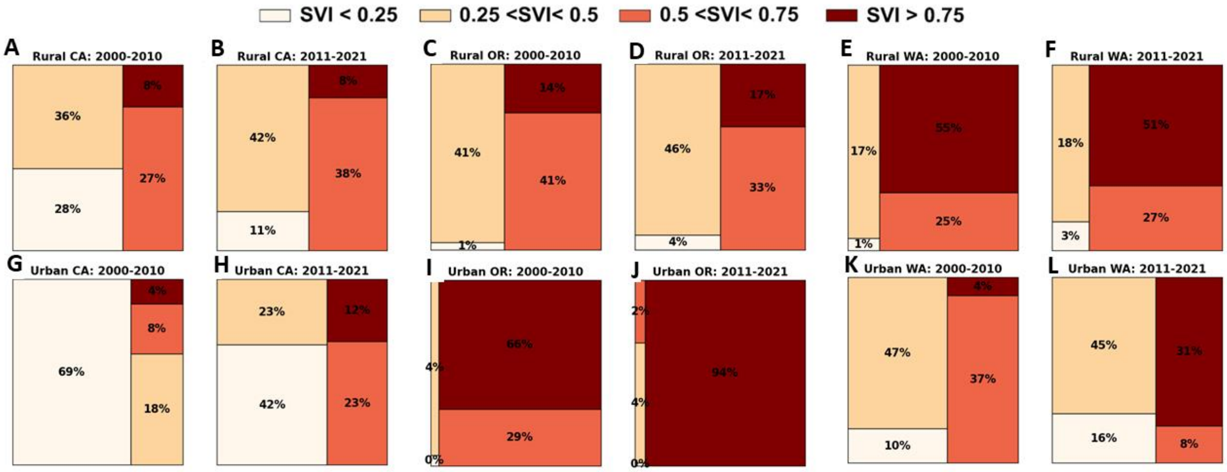

Figure S10. Percentage of exposures to fire by social vulnerability class from 2000-2010 (left) and 2011-2021 (right) in rural (top) and urban (bottom) areas in California (A,B,G,H), Oregon (C,D,I,J), and Washington (E,F,K,L).

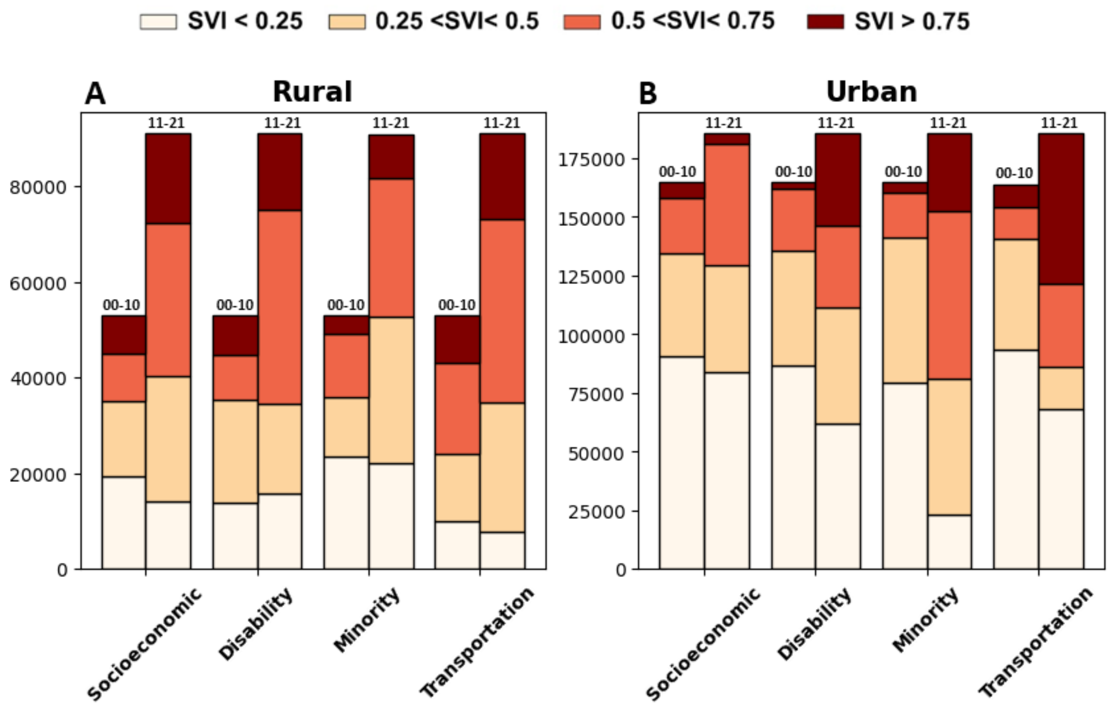

Figure S11. Decadal number of exposed people in four social vulnerability classes and each social vulnerability dimension in rural (left; A) and urban (right; B) areas across the study region. Note: y-axis limits differ by a factor 2, reflecting greater exposure of urban populations. See Fig. S1 for details of social vulnerability dimensions.

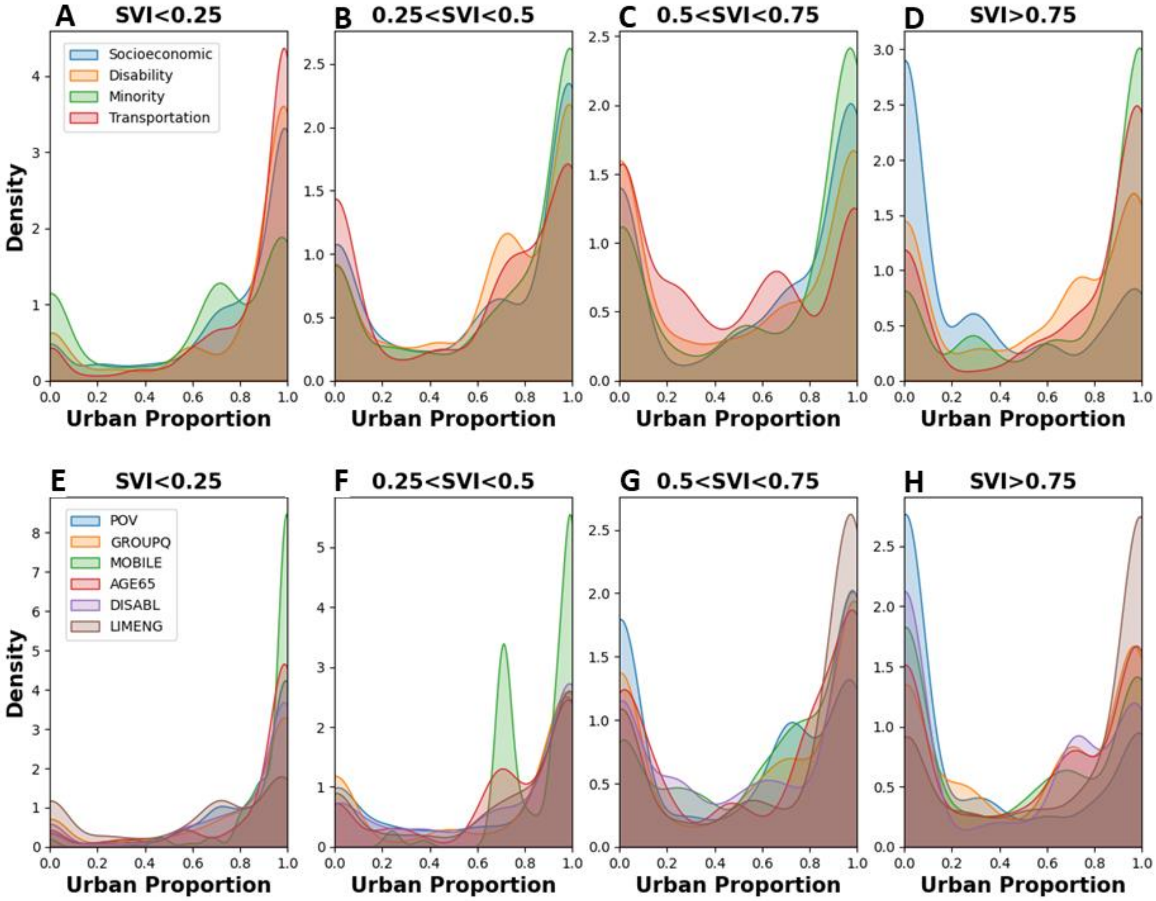

Figure S12. Urban proportion distribution for the exposed population in each social vulnerability class and social vulnerability dimension (top; A-D) and subdimension (bottom; E-H) across the study region. Urban proportion is defined as the proportion of urban residents in each census tract.

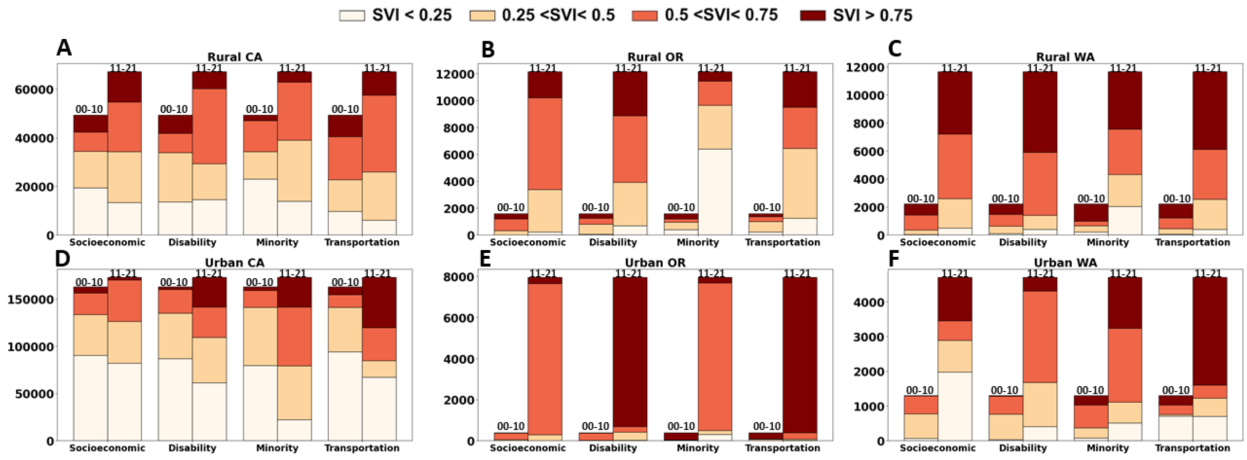

Figure S13. Decadal number of exposed people in four social vulnerability classes and each social vulnerability dimension in rural (top) and urban (bottom) areas in California (A,D), Oregon (B,E), and Washington (C,F). See Fig. S1 for details of social vulnerability dimensions.

Table S1. Exposures of Californians to fire from 2000-2021 by county and vulnerability class, and associated percentage of county population. Percentages are cumulative exposure from 2000-2021 normalized by the average annual population of the county.

County	Low vulnerability		Medium-low vulnerability		Medium-high vulnerability		High vulnerability	
	Exposure	% of population	Exposure	% of population	Exposure	% of population	Exposure	% of population
Los Angeles	62375	0.64	23325	0.24	8786	0.09	7207	0.07
Ventura	23841	2.88	12759	1.54	3345	0.40	7153	0.86
Butte	238	0.11	8331	3.77	28690	12.98	6919	3.13
Lake	0	0.00	126	0.19	3674	5.64	4998	7.68
San Diego	54860	1.77	24839	0.80	11040	0.36	3557	0.11
Riverside	1981	0.09	3833	0.17	2216	0.10	1282	0.06
Siskiyou	0	0.00	49	0.11	1409	3.14	944	2.10
San Bernardino	15003	0.73	6120	0.30	9556	0.46	932	0.05
Shasta	36	0.02	6651	3.73	3026	1.70	591	0.33
Trinity		0.00	0	0.00	1875	13.54	470	3.39
Mariposa	58	0.32	800	4.39	595	3.27	453	2.49
Yuba	0	0.00	1562	2.13	247	0.34	376	0.51
Yolo	0	0.00	0	0.00	397	0.19	341	0.17
Monterey	754	0.18	622	0.15	126	0.03	260	0.06
Kern	136	0.02	247	0.03	3787	0.44	251	0.03
Glenn	0	0.00	0	0.00	0	0.00	170	0.60
Mendocino	0	0.00	11	0.01	1561	1.78	124	0.14
Tehama	0	0.00	0	0.00	354	0.55	118	0.18
Fresno	4	0.00	1803	0.19	67	0.01	97	0.01
Plumas	2	0.01	2252	11.27	118	0.59	95	0.48
Colusa	0	0.00	0	0.00	70	0.32	67	0.31
Alpine	0	0.00	0	0.00	0	0.00	64	5.46
Del Norte	0	0.00	0	0.00	5	0.02	35	0.12
Tulare	0	0.00	71	0.02	584	0.13	35	0.01

Humboldt	3	0.00	0	0.00	141	0.10	21	0.02
Imperial	0	0.00	0	0.00	8	0.00	21	0.01
Lassen	34	0.10	1112	3.18	721	2.06	17	0.05
Sonoma	10888	2.24	8370	1.73	4331	0.89	17	0.00
Stanislaus	0	0.00	19	0.00	1	0.00	13	0.00
Tuolumne	0	0.00	418	0.77	68	0.12	8	0.01
Modoc	0	0.00	4	0.04	72	0.74	5	0.05
Sutter	0	0.00	0	0.00	1	0.00	4	0.00
Merced	0	0.00	0	0.00	1	0.00	3	0.00
San Luis Obispo	130	0.05	365	0.14	22	0.01	2	0.00
Inyo	12	0.06	127	0.68	8	0.04	1	0.01
Alameda	387	0.03	0	0.00	1	0.00	0	0.00
Contra Costa	93	0.01	784	0.07	15	0.00	0	0.00
El Dorado	1038	0.57	2026	1.11	65	0.04	0	0.00
Marin	26	0.01	0	0.00	0	0.00	0	0.00
Napa	1487	1.08	3906	2.83	1487	1.08	0	0.00
Orange	26396	0.88	162	0.01	0	0.00	0	0.00
Placer	467	0.13	1340	0.37	20	0.01	0	0.00
Sacramento	364	0.03	3	0.00	0	0.00	0	0.00
San Francisco	0	0.00	0	0.00	0	0.00	0	0.00
San Joaquin	0	0.00	46	0.01	6	0.00	0	0.00
San Mateo	0	0.00	0	0.00	0	0.00	0	0.00
Santa Clara	398	0.02	22	0.00	122	0.01	0	0.00
Santa Cruz	230	0.09	340	0.13	5	0.00	0	0.00
Solano	2361	0.57	100	0.02	104	0.03	0	0.00
Kings	0	0.00	0	0.00	0	0.00	0	0.00
Santa Barbara	3341	0.79	1061	0.25	54	0.01	0	0.00
Amador	0	0.00	153	0.40	51	0.13	0	0.00
Madera	0	0.00	542	0.35	440	0.29	0	0.00

Nevada	313	0.32	2	0.00	304	0.31	0	0.00
San Benito	0	0.00	23	0.04	9	0.02	0	0.00
Calaveras	50	0.11	749	1.63	2889	6.28	0	0.00
Mono	236	1.65	448	3.13	418	2.92	0	0.00
Sierra	0	0.00	6	0.18	5	0.15	0	0.00

Table S2. Exposures of Oregonians to fire from 2000-2021 by county and vulnerability class, and associated percentage of county population. Percentages are cumulative exposure from 2000-2021 normalized by the average annual population of the county.

County	Low vulnerability		Medium-low vulnerability		Medium-high vulnerability		High vulnerability	
	Exposure	% of population	Exposure	% of population	Exposure	% of population	Exposure	% of population
Jackson	10	0.00	927	0.45	309	0.15	7191	3.51
Jefferson	0	0.00	14	0.06	137	0.62	1399	6.38
Wasco	0	0.00	298	1.18	859	3.40	501	1.98
Hood River	0	0.00	0	0.00	162	0.72	343	1.53
Umatilla	0	0.00	2	0.00	25	0.03	254	0.33
Klamath	1	0.00	18	0.03	93	0.14	185	0.28
Malheur	0	0.00	0	0.00	128	0.41	36	0.12
Curry	0	0.00	20	0.09	13	0.06	33	0.15
Josephine	0	0.00	252	0.30	357	0.43	31	0.04
Douglas	0	0.00	24	0.02	689	0.64	19	0.02
Lake	0	0.00	0	0.00	99	1.25	18	0.23
Harney	0	0.00	39	0.53	28	0.38	2	0.03
Baker	0	0.00	154	0.96	56	0.35	1	0.01
Clackamas	465	0.12	861	0.23	18	0.00	0	0.00
Deschutes	25	0.02	7	0.00	19	0.01	0	0.00
Lane	3	0.00	1712	0.48	11	0.00	0	0.00

Marion	0	0.00	2049	0.65	7	0.00	0	0.00
Multnomah	0	0.00	39	0.01	0	0.00	0	0.00
Union	0	0.00	4	0.02	0	0.00	0	0.00
Clatsop	0	0.00	0	0.00	0	0.00	0	0.00
Linn	0	0.00	8	0.01	564	0.48	0	0.00
Tillamook	0	0.00	0	0.00	0	0.00	0	0.00
Washington	2	0.00	0	0.00	0	0.00	0	0.00
Yamhill	0	0.00	0	0.00	0	0.00	0	0.00
Benton	0	0.00	0	0.00	0	0.00	0	0.00
Lincoln	0	0.00	0	0.00	1023	2.23	0	0.00
Polk	0	0.00	0	0.00	0	0.00	0	0.00
Columbia	0	0.00	0	0.00	0	0.00	0	0.00
Coos	0	0.00	0	0.00	0	0.00	0	0.00
Morrow	0	0.00	0	0.00	1	0.01	0	0.00
Crook	0	0.00	7	0.03	8	0.04	0	0.00
Gilliam	0	0.00	28	1.50	42	2.25	0	0.00
Wallowa	0	0.00	54	0.77	42	0.60	0	0.00
Grant	0	0.00	2	0.03	213	2.87	0	0.00
Wheeler	0	0.00	26	1.81	32	2.23	0	0.00
Sherman	0	0.00	23	1.31	35	1.99	0	0.00

Table S3. Exposures of Washingtonians to fire from 2000-2021 by county and vulnerability class, and associated percentage of county population. Percentages are cumulative exposure from 2000-2021 normalized by the average annual population of the county.

County	Low vulnerability		Medium-low vulnerability		Medium-high vulnerability		High vulnerability	
	Exposure	% of population	Exposure	% of population	Exposure	% of population	Exposure	% of population
Okanogan	38	0.09	770	1.87	1596	3.87	3956	9.60
Chelan	759	1.04	1019	1.40	606	0.83	1618	2.22
Douglas	0	0.00	69	0.18	155	0.40	1264	3.25
Yakima	0	0.00	599	0.24	357	0.15	466	0.19
Grant	0	0.00	52	0.06	90	0.10	381	0.42
Ferry	0	0.00	0	0.00	72	0.95	344	4.54
Stevens	97	0.22	163	0.37	235	0.54	202	0.46
Benton	23	0.01	1667	0.94	310	0.17	150	0.08
Klickitat	0	0.00	46	0.23	170	0.83	114	0.56
Adams	0	0.00	0	0.00	4	0.02	105	0.56
Mason	0	0.00	23	0.04	1	0.00	43	0.07
Spokane	116	0.02	122	0.03	68	0.01	39	0.01
Clark	0	0.00	0	0.00	0	0.00	12	0.00
Walla Walla	0	0.00	19	0.03	9	0.02	7	0.01
Franklin	0	0.00	1	0.00	326	0.39	3	0.00
Grays	0	0.00	0	0.00	0	0.00	0	0.00

Harbor								
King	0	0.00	0	0.00	0	0.00	0	0.00
Kitsap	0	0.00	0	0.00	0	0.00	0	0.00
Pacific	0	0.00	0	0.00	0	0.00	0	0.00
Pierce	1	0.00	0	0.00	0	0.00	0	0.00
San Juan	0	0.00	0	0.00	0	0.00	0	0.00
Skagit	0	0.00	0	0.00	0	0.00	0	0.00
Snohomish	0	0.00	3	0.00	0	0.00	0	0.00
Thurston	0	0.00	0	0.00	0	0.00	0	0.00
Whatcom	0	0.00	0	0.00	14	0.01	0	0.00
Whitman	8	0.02	0	0.00	334	0.74	0	0.00
Lewis	0	0.00	2	0.00	0	0.00	0	0.00
Island	0	0.00	0	0.00	0	0.00	0	0.00
Kittitas	216	0.52	349	0.84	1	0.00	0	0.00
Cowlitz	0	0.00	0	0.00	0	0.00	0	0.00
Pend Oreille	0	0.00	0	0.00	0	0.00	0	0.00
Skamania	0	0.00	37	0.33	0	0.00	0	0.00
Clallam	0	0.00	0	0.00	0	0.00	0	0.00
Lincoln	0	0.00	271	2.56	32	0.30	0	0.00
Asotin	56	0.26	17	0.08	0	0.00	0	0.00
Garfield	7	0.31	7	0.31	16	0.71	0	0.00

Jefferson	0	0.00	0	0.00	2	0.01	0	0.00
Columbia	0	0.00	0	0.00	219	5.37	0	0.00
Wahkiakum	0	0.00	0	0.00	0	0.00	0	0.00

Table S4. Representative statistics (median and interquartile range [IQR]) of distributions of the social vulnerability index among California's state population (all) and fire-exposed population (exposed). We used Mood's median test to assess whether median vulnerability of the two distributions was similar, and Bartlett test to assess whether the variance of the two distributions was similar (a proxy for similarity of IQR). We used a Mann-Whitney U test to assess whether the two distributions were similar. We set the significance level for all tests at 5% (95% confidence level).

Social vulnerability dimension	Median			IQR			Similar Distribution ?
	All	Exposed	p-value	All	Exposed	p-value	p-value
Overall SVI	0.60	0.28	0.0000	0.54	0.41	0.0000	0.0000
Socioeconomic Status	0.56	0.31	0.0000	0.56	0.41	0.0000	0.0000
Household Composition & Minority	0.53	0.32	0.0000	0.52	0.40	0.0000	0.0000
Minority Status & Language	0.70	0.39	0.0000	0.47	0.35	0.0000	0.0000
Housing Type & Transportation	0.57	0.37	0.0000	0.50	0.50	0.4131	0.0000
Group quarters	0.44	0.43	0.6984	0.72	0.76	0.0036	0.1723
Below poverty	0.50	0.31	0.0000	0.49	0.37	0.0000	0.0000
Speaks English less than well	0.73	0.37	0.0000	0.41	0.41	0.0000	0.0000
Disability	0.37	0.34	0.0062	0.40	0.49	0.0000	0.2145
Age 65 or older	0.31	0.55	0.0000	0.44	0.68	0.0000	0.0000
Mobile home	0.42	0.64	0.0000	0.64	0.82	0.1344	0.0000

Table S5. Representative statistics (median and interquartile range [IQR]) of distributions of the social vulnerability index among Oregon's state population (all) and fire-exposed population (exposed). We used Mood's median test to assess whether median vulnerability of the two distributions was similar, and Bartlett test to assess whether the variance of the two distributions was similar (a proxy for similarity of IQR). We used a Mann-Whitney U test to assess whether the two distributions were similar. We set the significance level for all tests at 5% (95% confidence level).

Social vulnerability dimension	Median			IQR			Similar Distribution ?
	All	Exposed	p-value	All	Exposed	p-value	p-value
Overall SVI	0.55	0.70	0.0000	0.42	0.43	0.0000	0.0000
Socioeconomic Status	0.50	0.63	0.0000	0.40	0.20	0.0000	0.0000
Household Composition & Minority	0.51	0.71	0.0000	0.47	0.33	0.0000	0.0000
Minority Status & Language	0.53	0.50	0.7764	0.46	0.47	0.0000	0.0000
Housing Type & Transportation	0.60	0.73	0.0000	0.45	0.39	0.0000	0.0000
Group quarters	0.58	0.50	0.0000	0.75	0.83	0.7213	0.4164
Below poverty	0.54	0.68	0.0000	0.36	0.27	0.0000	0.0000
Speaks English less than well	0.58	0.49	0.0000	0.44	0.71	0.0000	0.0000
Disability	0.53	0.81	0.0000	0.45	0.21	0.0000	0.0000
Age 65 or older	0.52	0.85	0.0000	0.48	0.16	0.0000	0.0000
Mobile home	0.70	0.91	0.0000	0.38	0.07	0.0000	0.0000

Table S6. Representative statistics (median and interquartile range [IQR]) of distributions of the social vulnerability index among Washington's state population (all) and fire-exposed population (exposed). We used Mood's median test to assess whether median vulnerability of the two distributions was similar, and Bartlett test to assess whether the variance of the two distributions was similar (a proxy for similarity of IQR). We used a Mann-Whitney U test to assess whether the two distributions were similar. We set the significance level for all tests at 5% (95% confidence level).

Social vulnerability dimension	Median			IQR			Similar Distribution?
	All	Exposed	p-value	All	Exposed	p-value	p-value
Overall SVI	0.44	0.67	0.0000	0.45	0.42	0.0000	0.0000
Socioeconomic Status	0.40	0.66	0.0000	0.41	0.38	0.0140	0.0000
Household Composition & Minority	0.47	0.66	0.0000	0.45	0.30	0.0000	0.0000
Minority Status & Language	0.54	0.64	0.0000	0.45	0.31	0.0000	0.0000
Housing Type & Transportation	0.53	0.73	0.0000	0.49	0.37	0.0000	0.0000
Group quarters	0.48	0.68	0.0000	0.74	0.27	0.0000	0.0000
Below poverty	0.43	0.68	0.0000	0.39	0.38	0.3110	0.0000
Speaks English less than well	0.57	0.66	0.0000	0.43	0.35	0.7263	0.0000
Disability	0.45	0.54	0.0000	0.44	0.43	0.0000	0.0000
Age 65 or older	0.39	0.66	0.0000	0.43	0.41	0.0000	0.0000
Mobile home	0.65	0.89	0.0000	0.36	0.11	0.0000	0.0000

Table S7. Representative statistics (median and interquartile range, IQR) of distributions of dimensions of the social vulnerability index for the exposed population in the counterfactual scenario versus observed conditions. We used Mood's median test to assess whether median vulnerability of the two distributions was similar, and Bartlett test to assess whether the variance of the two distributions was similar (a proxy for similarity of IQR). We used a Mann-Whitney U test to assess whether the two distributions were similar. We set the significance level for all tests at 5% (95% confidence level).

	**Social Vulnerability Dimension**	**Median**			**IQR**			**Similar Distribution?**
		**Obs.**	**Scen.**	**p-value**	**Obs.**	**Scen.**	**p-value**	**p-value**
**CA**	Socioeconomic Status	0.37	0.37	0.7927	0.39	0.56	0.0000	0.0039
	Household Composition & Minority	0.36	0.36	0.2434	0.42	0.53	0.0000	0.0000
	Minority Status & Language	0.50	0.15	0.0000	0.32	0.32	0.0000	0.0000
	Housing Type & Transportation	0.54	0.38	0.0000	0.56	0.41	0.0000	0.0000
**OR**	Socioeconomic Status	0.63	0.65	0.0000	0.20	0.14	0.0009	0.0000
	Household Composition & Minority	0.76	0.74	0.0000	0.27	0.23	0.0000	0.0000
	Minority Status & Language	0.50	0.61	0.0000	0.47	0.40	0.0000	0.0000
	Housing Type & Transportation	0.76	0.64	0.0000	0.39	0.56	0.0000	0.0000
**WA**	Socioeconomic Status	0.66	0.69	0.0000	0.39	0.30	0.0000	0.0000
	Household Composition & Minority	0.67	0.62	0.0000	0.30	0.36	0.1048	0.0000
	Minority Status & Language	0.61	0.64	0.0000	0.31	0.48	0.0000	0.0000
	Housing Type & Transportation	0.78	0.75	0.0000	0.33	0.43	0.3319	0.0000

Table S8. Representative statistics (median and interquartile range, IQR) of distributions of subdimensions of the social vulnerability index for the exposed population in the counterfactual scenario versus observed conditions. We used Mood's median test to assess whether median vulnerability of the two distributions was similar, and Bartlett test to assess whether the variance of the two distributions was similar (a proxy for similarity of IQR). We used a Mann-Whitney U test to assess whether the two distributions were similar. We set the significance level for all tests at 5% (95% confidence level).

	Social Vulnerability Subdimension	Median			IQR			Similar Distribution ?
		Obs.	Scen.	p-value	Obs.	Scen.	p-value	p-value
CA	Group quarters	0.61	0.00	0.0000	0.77	0.78	0.0000	0.0000
	Below poverty	0.33	0.38	0.0000	0.33	0.41	0.0000	0.0000
	Speaks English less than well	0.54	0.19	0.0000	0.35	0.30	0.0000	0.0000
	Disability	0.51	0.34	0.0000	0.55	0.60	0.0000	0.0000
	Age 65 or older	0.81	0.58	0.0000	0.48	0.63	0.0000	0.0000
	Mobile home	0.69	0.70	0.0000	0.84	0.40	0.0000	0.0000
OR	Group quarters	0.50	0.64	0.0000	0.85	0.71	0.0000	0.0000
	Below poverty	0.68	0.58	0.0000	0.27	0.11	0.0000	0.0000
	Speaks English less than well	0.41	0.63	0.0000	0.71	0.41	0.0000	0.0000
	Disability	0.81	0.60	0.0000	0.17	0.20	0.0000	0.0000
	Age 65 or older	0.90	0.77	0.0000	0.16	0.17	0.2628	0.0000
	Mobile home	0.92	0.93	0.0000	0.07	0.10	0.0000	0.0000
WA	Group quarters	0.69	0.74	0.0000	0.24	0.75	0.0000	0.0000

	Below poverty	0.69	0.68	0.0000	0.37	0.32	0.0000	0.0000
	Speaks English less than well	0.64	0.72	0.0000	0.47	0.43	0.0000	0.0000
	Disability	0.54	0.48	0.0000	0.45	0.24	0.0000	0.0000
	Age 65 or older	0.66	0.47	0.0000	0.42	0.28	0.0000	0.0000
	Mobile home	0.90	0.90	0.1809	0.11	0.11	0.0000	0.0000

Supplemental Data 1. Census-tract level total population, social vulnerability index, population exposure to fire, and proportion of residents that were urban in 2000 and 2001 in California, Oregon and Washington.
Supplemental Data 2. Census-tract level total population, social vulnerability index, population exposure to fire, and proportion of residents that were urban in 2002 and 2003 in California, Oregon and Washington.
Supplemental Data 3. Census-tract level total population, social vulnerability index, population exposure to fire, and proportion of residents that were urban in 2004 and 2005 in California, Oregon and Washington.
Supplemental Data 4. Census-tract level total population, social vulnerability index, population exposure to fire, and proportion of residents that were urban in 2006 and 2007 in California, Oregon and Washington.
Supplemental Data 5. Census-tract level total population, social vulnerability index, population exposure to fire, and proportion of residents that were urban in 2008 and 2009 in California, Oregon and Washington.
Supplemental Data 6. Census-tract level total population, social vulnerability index, population exposure to fire, and proportion of residents that were urban in 2010 and 2011 in California, Oregon and Washington.
Supplemental Data 7. Census-tract level total population, social vulnerability index, population exposure to fire, and proportion of residents that were urban in 2012 and 2013 in California, Oregon and Washington.
Supplemental Data 8. Census-tract level total population, social vulnerability index, population exposure to fire, and proportion of residents that were urban in 2014 and 2015 in California, Oregon and Washington.
Supplemental Data 9. Census-tract level total population, social vulnerability index, population exposure to fire, and proportion of residents that were urban in 2016 and 2017 in California, Oregon and Washington.
Supplemental Data 10. Census-tract level total population, social vulnerability index, population exposure to fire, and proportion of residents that were urban in 2018 and 2019 in California, Oregon and Washington.
Supplemental Data 11. Census-tract level total population, social vulnerability index, population exposure to fire, and proportion of residents that were urban in 2020 and 2021 in California, Oregon and Washington.
